# Low-Temperature Synthesis of Highly Preferentially Oriented ε-Ga_2_O_3_ Films for Solar-Blind Detector Application

**DOI:** 10.3390/nano15241867

**Published:** 2025-12-12

**Authors:** He Tian, Yijun Zhang, Hong Wang, Daogui Liao, Jiale Di, Chao Liu, Wei Ren, Zuo-Guang Ye

**Affiliations:** 1 School of Electronic Science and Engineering, Electronic Materials Research Laboratory, Key Laboratory of the Ministry of Education, State Key Laboratory for Manufacturing Systems Engineering Xi’an Jiaotong University, Xi’an 710049, Chinadgliao@stu.xjtu.edu.cn (D.L.);; 2 State Key Laboratory for Manufacturing Systems Engineering & The International Joint Laboratory for Micro/Nano Manufacturing and Measurement Technology, Xi’an Jiaotong University, Xi’an 710049, China; 3 Department of Chemistry and 4D LABS, Simon Fraser University, Burnaby, BC V5A 1S6, Canada

**Keywords:** ε-Ga_2_O_3_, Atomic Layer Deposition, Solar-Blind Photodetector

## Abstract

As one of the polymorphs of the gallium oxide family, ε gallium oxide (ε-Ga_2_O_3_) demonstrates promising potential in high-power electronic devices and solar-blind photodetection applications. However, the synthesis of pure-phase ε-Ga_2_O_3_ remains challenging through low-energy consumption methods, due to its metastable phase of gallium oxide. In this study, we have fabricated pure-phase and highly oriented ε-Ga_2_O_3_ thin films on c-plane sapphire substrates via thermal atomic layer deposition (ALD) at a low temperature of 400 °C, utilizing low-reactive trimethylgallium (TMG) as the gallium precursor and ozone (O_3_) as the oxygen source. X-ray diffraction (XRD) results revealed that the in situ-grown ε-Ga_2_O_3_ films exhibit a preferred orientation parallel to the (002) crystallographic plane, and the pure ε phase remains stable following a post-annealing up to 800 °C, but it completely transforms into β-Ga_2_O_3_ once the thermal treatment temperature reaches 900 °C. Notably, post-annealing at 800 °C significantly enhanced the crystalline quality of ε-Ga_2_O_3_. To evaluate the optoelectronic characteristics, metal–semiconductor–metal (MSM)-structured solar-blind photodetectors were fabricated using the ε-Ga_2_O_3_ films. The devices have an extremely low dark current (<1 pA), a high photo-to-dark current ratio (>10^6^), a maximum responsivity (>1 A/W), and the optoelectronic properties maintained stability under varying illumination intensities. This work provides valuable insights into the low-temperature synthesis of high-quality ε-Ga_2_O_3_ films and the development of ε-Ga_2_O_3_-based solar-blind photodetectors for practical applications.

## 1. Introduction

The realization of high-performance power electronic devices in such applications as electric vehicle charging, grid-scale energy storage, and improvements in high-speed trains and other systems depends heavily on wide-bandgap semiconductor materials [1,2,3]. Owing to its high breakdown field, ultra-high Baliga’s figure of merit (BFOM), wide bandgap, excellent thermal stability, and chemical inertness, Ga_2_O_3_ has emerged as one of the most promising materials beyond the third-generation semiconductors of silicon carbide (SiC) and gallium nitride (GaN) [4,5,6,7,8]. It has attracted significant attention in the fields of power devices and solar-blind ultraviolet detection [9,10,11,12,13,14]. To date, at least five crystalline polymorphs of gallium oxide have been reported: β, α, δ, γ, and ε (also referred to as κ-phase). Among them, the extensively studied β-phase is the only thermodynamically stable form. At high temperatures, all the other metastable phases transform irreversibly into the β-phase. Their stability follows the order: β < ε(κ) < α < γ < δ. Notably, ε-Ga_2_O_3_ exhibits an orthorhombic, pseudo-hexagonal structure with rotational nanodomains of gallium oxide (also defined as κ-Ga_2_O_3_) [15,16,17].

Among the various polymorphs of gallium oxide, the ε(κ)-phase is the second most stable phase at atmospheric pressure, and it transforms into β-Ga_2_O_3_ when the post-annealing temperature reaches 800–1000 °C [18]. Therefore, devices based on ε-Ga_2_O_3_ must be kept below 800 °C throughout both the fabrication process and operational applications [19]. Moreover, among the metastable phases of gallium oxide, only the ε-phase possesses a non-centrosymmetric and polar structure. Based on a combination of first-principle calculations [20] and group theory, its spontaneous polarization is predicted to be approximately 23 μC/cm^2^. This property makes ε-Ga_2_O_3_ highly promising for ferroelectric semiconductor integrated devices. However, so far, there have been very few experimental reports on the ferroelectric properties of ε-Ga_2_O_3_ [21,22,23,24]. In addition, ε-Ga_2_O_3_ exhibits a significantly higher phase transition temperature (ε-to-β) compared to other metastable phases. Although compared to the stable β-Ga_2_O_3_, the metastable ε-Ga_2_O_3_ exhibits a higher symmetry and a smaller lattice mismatch with sapphire substrates, and [25,26,27,28,29] growing pure-phase ε-Ga_2_O_3_ thin films is considerably more challenging than growing β-Ga_2_O_3_ films. These difficulties arise from several key factors, which are summarized in Table 1 below.

**Figure 1:**
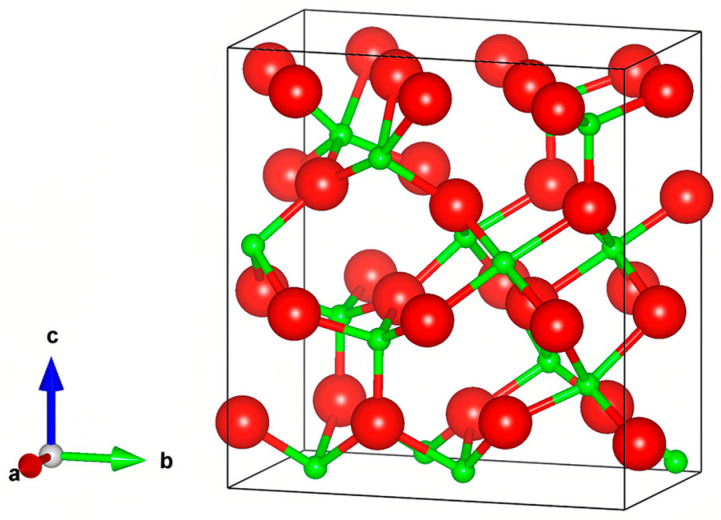
The crystal structure of ε-Ga_2_O_3_.

Currently, ε-Ga_2_O_3_ thin films are primarily fabricated at high temperatures (>500 °C) using such methods as MOCVD [30,31,32], MOPVE [33], PLD, and custom-built ALD systems [16], with film thicknesses typically ranging from several hundred nanometers to a few micrometers [34,35]. In this study, the ε-Ga_2_O_3_ film with an orthogonal structure (as showing in Figure 1) were deposited on sapphire substrates using a standard commercial ALD system at relatively low temperatures. Thinner films enable more precise control, resulting in improved energy conversion efficiency, enhanced sensing sensitivity, and higher-resolution optical imaging, in such applications as electronics, optics, and sensors.

## 2. Experimental Section

### 2.1. Thin Film Fabrication and Characterization

ε-Ga_2_O_3_ thin films were deposited using a commercial ALD (PICOSUN R200 Advanced, Helsinki, Finland) system. The (001)-oriented ε-Ga_2_O_3_ films were grown on commercially available (0006)-oriented sapphire substrates via a thermal ALD process. Prior to deposition, the sapphire substrates were cleaned in acetone, ethanol, and deionized water for 10 min each by sonication, followed by drying with nitrogen gas. Trimethylgallium (TMG) and ozone (O_3_) were used as the gallium and oxygen precursors, respectively. The TMG was stored in a cylinder at room temperature. During the deposition, the TMG pulse and purge times were set to 0.1 s and 6 s, respectively, while the ozone pulse and purge times were 4 s and 5 s, respectively. High-purity nitrogen was employed as the carrier gas at a flow rate of 200 sccm. A total of 1000 cycles of gallium oxide thin films were grown at 250 °C, 300 °C, 350 °C, and 400 °C, respectively. Each cycle consisted of the following sequence: TMG pulse, TMG purge, ozone pulse, and ozone purge. Selected as-deposited films were subsequently annealed at different temperatures for 2 h. The film morphology was characterized by atomic force microscopy (AFM, Dimension Icon, Bruker, Billerica, MA, USA). The microstructure and thickness were analyzed using X-ray diffraction (XRD, Rigaku, Tokyo, Japan) and X-ray reflectivity (XRR), respectively. Optical transmittance was measured by UV–Visible spectrophotometry.

### 2.2. Device Preparation and Testing

Metal–semiconductor–metal (MSM) solar-blind photodetectors were fabricated via photolithography, followed by sputtering of two gold electrodes. The resulting interdigitated fork-finger electrodes possess a finger length of 0.2 mm, a finger width of 32 μm, an inter-finger pitch of 8 μm, a unilateral electrode area of 0.2 mm^2^, and an effective illumination area of 0.021 mm^2^. The optoelectronic performance of the photodetectors was characterized using a semiconductor parameter analyzer (Keithley 4200A-SCS, Beaverton, OR, USA).

## 3. Results and Discussion

**Figure 2:**
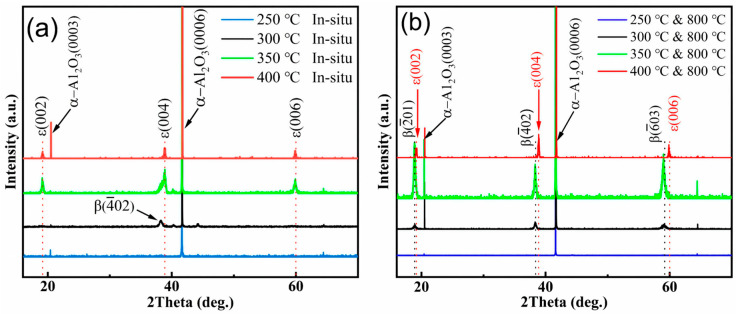
XRD patterns of (**a**) Ga_2_O_3_ films as-grown in situ at 250 °C, 300 °C, 350 °C, and 400 °C; (**b**) after annealing at 800 °C for 2 h.

Figure 2 presents the XRD patterns of the Ga_2_O_3_ thin films deposited on c-plane sapphire substrates at different temperatures. As shown in Figure 2a, no diffraction peaks other than the sapphire (006) peak are observed for the Ga_2_O_3_ film grown at 250 °C, indicating an amorphous structure. When the deposition temperature was increased to 300 °C, a diffraction peak emerged at 38.4°, which corresponds to the ($\bar{4}$02) plane of the β-phase, suggesting the initial formation of β-Ga_2_O_3_ at this temperature. When the deposition temperature was further increased to 350 °C, strong diffraction peaks were observed at 19.18°, 38.88°, and 60°, corresponding to the (002), (004), and (006) crystallographic planes of the ε-phase, respectively. However, due to the close spacings of the β-phase ($\bar{4}$02) diffraction peak from the ε-phase (004) peak and the apparent asymmetry of the ε-phase (004) peak with a shoulder on the left side, it is reasonable to suspect the presence of β-phase with its ($\bar{4}$02) peak at 38.4° in the sample deposited at 350 °C. When the deposition temperature was increased to 400 °C, only three sharp diffraction peaks corresponding to the ε-phase were observed, indicating the formation of pure ε-Ga_2_O_3_.

The above Ga_2_O_3_ films grown on the sapphire substrate were post-annealed in a tube furnace at 800 °C in an air atmosphere for 2 h, and the XRD patterns of the films are shown in Figure 2b. The film deposited at 250 °C remained amorphous even after annealing. Those grown at 300 °C and 350 °C exhibited distinct diffraction peaks at 18.9°, 38.4°, and 59.2° after post-annealing, which are indexed to the β-phase ($\bar{2}$01), ($\bar{4}$02), and ($\bar{6}$03) planes, respectively, indicating a complete transformation to the stable β-phase. In contrast, only the film grown at 400 °C remained in the ε-phase after post-annealing at 800 °C, demonstrating that pure ε-phase exhibits better thermal stability than the mixed phases grown at 350 °C. The sharpening of the diffraction peaks after annealing suggests an improved crystallinity of ε-Ga_2_O_3_. However, the films with mixed phases undergo the ε-phase transformation to the β-plane in the post-annealing process.

**Figure 3:**
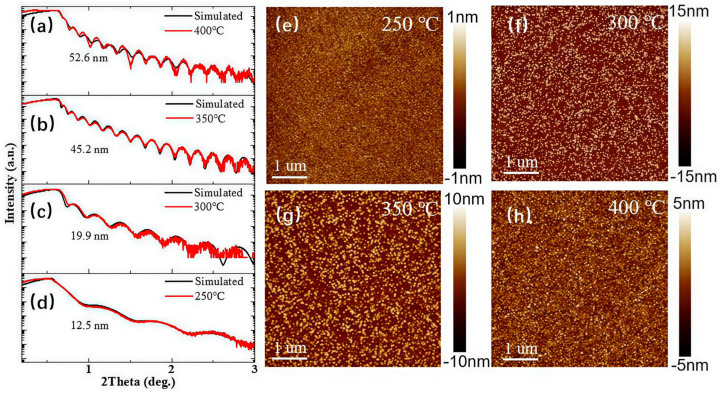
The XRR results and their fitting curves of the Ga_2_O_3_ films grown in situ at temperatures of (**a**) 250 °C, (**b**) 300 °C, (**c**) 350 °C, and (**d**) 400 °C. The AFM micrographs of the Ga_2_O_3_ films grown at temperatures of (**e**) 250 °C, (**f**) 300 °C, (**g**) 350 °C, and (**h**) 400 °C.

Figure 3 presents the XRR measurement results and AFM images of the gallium oxide thin films deposited at different temperatures. Figure 3a–d display the XRR results and corresponding fitting curves for the films grown at 250 °C, 300 °C, 350 °C, and 400 °C, respectively. The fitted thicknesses are 12.5 nm, 19.9 nm, 45.2 nm, and 52.6 nm, with growth rates of 0.125 Å/cycle, 0.199 Å/cycle, 0.452 Å/cycle, and 0.526 Å/cycle, respectively. The growth rate increases with temperature, showing a significant acceleration between 300 °C and 350 °C.

Figure 3e–h show the surface morphology of the gallium oxide films grown in situ at 250 °C, 300 °C, 350 °C, and 400 °C, respectively. The film grown at 250 °C exhibited a relatively smooth surface with a roughness of 0.291 nm. The Ga_2_O_3_ thin film deposited at 300 °C exhibited a morphology composed of densely packed columns. This structure originates from two primary factors: the transition from an amorphous phase formed at 250 °C to the crystalline β-phase gallium oxide at 300 °C, combined with the inherent tendency of the β-Ga_2_O_3_ to grow in a columnar manner. As a result, the surface roughness increased to 5.3 nm. When the temperature increases to 350 °C, the film also exhibited a granular morphology, with the grain size increasing noticeably compared to that at 300 °C, while the surface roughness decreased to 2.96 nm. At 400 °C, the higher substrate temperature promoted atomic migration fomr a smoother and denser film surface. As a result, the surface roughness was further reduced to 1.52 nm, reflecting an improved morphological uniformity.

**Figure 4:**
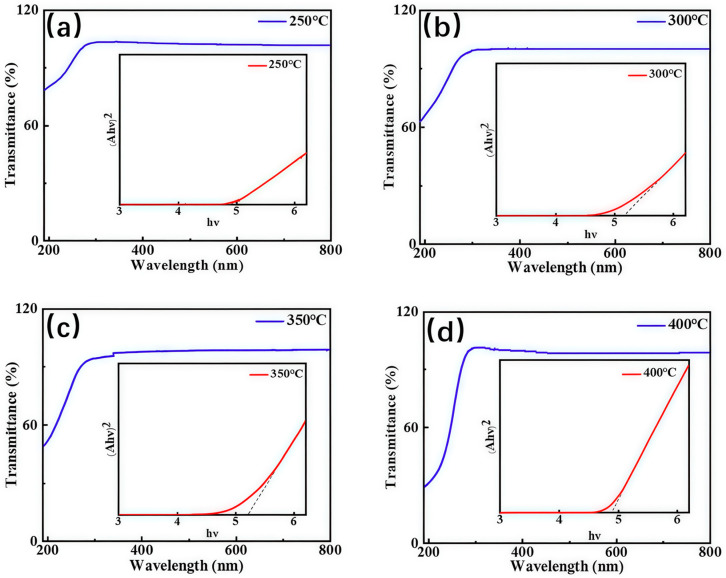
The transmittance spectra of the Ga_2_O_3_ films grown in situ at temperatures of (**a**) 250 °C, (**b**) 300 °C, (**c**) 350 °C, and (**d**) 400 °C, along with the corresponding curves of (αhν)2 versus hν (insets).

Figure 4 shows the transmittance curves and the corresponding plots of (αhν)^2^ versus photon energy (hν) (insets) for the gallium oxide films deposited at various temperatures. The transmittance of the films exceeds 95% over the wavelength range of 300–800 nm. Furthermore, the absorption edge of the ε-Ga_2_O_3_ film grown at 400 °C is significantly steeper. The insets show the Tauc plots obtained from the absorption spectra, with (αhν)^2^ plotted as a function of hν. The optical band gaps of the films deposited at 250 °C, 300 °C, 350 °C, and 400 °C were determined to be 4.96 eV, 5.15 eV, 5.21 eV, and 4.89 eV, respectively.

**Figure 5:**
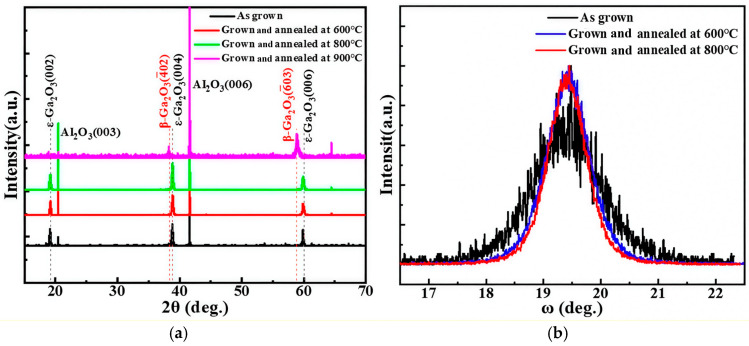
(**a**) The XRD patterns of ε-Ga_2_O_3_ films grown at 400 °C after annealing at different temperatures, and (**b**) the rocking curves of the (004) diffraction peak of ε-Ga_2_O_3_ films.

Figure 5a presents the XRD patterns of the as-grown pure-phase ε-Ga_2_O_3_ film along with those subjected to post-annealing at 600 °C, 800 °C, and 900 °C. The results demonstrate that the ε-Ga_2_O_3_ films maintained their phase purity after annealing at 600 °C and 800 °C, with no detectable secondary phases appeared or preferred orientation changed, indicating an excellent thermal stability. In contrast, after annealing at 900 °C, the film underwent a complete transition to the β-phase, exhibiting a strong preferential orientation parallel to the ($\bar{2}$01) plane.

To evaluate the crystalline quality of the ε-Ga_2_O_3_ films before and after post-annealing, the rocking curves of the (004) diffraction peak were measured for the as-grown samples and those annealed at 600 °C and 800 °C, as shown in Figure 5b. The diffraction peak narrows with increasing annealing temperature. The full widths at the half maximum (FWHM) values of the (004) peak are 0.86° for the as-grown film, 0.82° after annealing at 600 °C, and 0.73° after annealing at 800 °C, indicating a progressive improvement in the crystal quality with higher annealing temperature.

**Figure 6:**
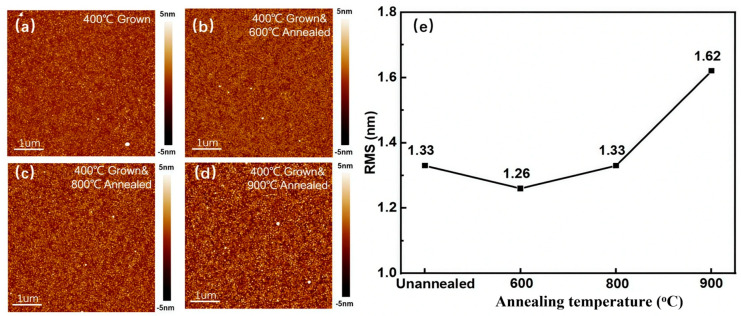
The AFM images (**a**–**d**) and surface roughness (**e**) of the ε-Ga_2_O_3_ films annealed at different temperatures.

The surface morphology of the as-grown and post-annealed Ga_2_O_3_ films was characterized by atomic force microscopy (AFM). The pristine ε-Ga_2_O_3_ film grown at 400 °C for 1000 cycles exhibits a relatively smooth surface and uniform particles (Figure 6e). Annealing at temperatures up to 800 °C induced no significant morphological changes. In contrast, annealing at 900 °C triggered noticeable grain growth, resulting in an increased root-mean-square (RMS) roughness of 1.62 nm, while maintaining an overall smooth surface. Given that the ε-Ga_2_O_3_ films grown at low temperatures in this work exhibit a good crystalline quality and the preferred orientation, a solar-blind photodetector was fabricated based on the film grown at 400 °C with 1000 cycles to explore its photoelectric properties. The photoresponse of the device to 254 nm ultraviolet (UV) light was characterized under different irradiation intensities. The current–voltage (I-V) curves were measured under light intensities ranging from 40 to 400 μW/cm^2^.

**Figure 7:**
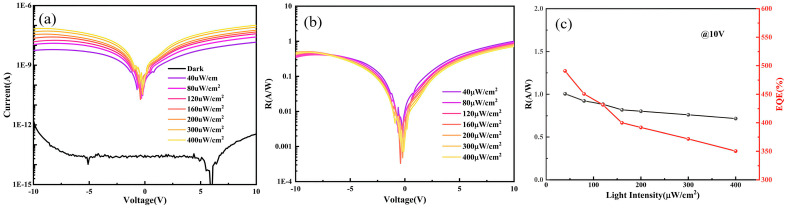
(**a**) The I-V characteristic curves of the ε-Ga_2_O_3_-based photodetectors under different light intensities. (**b**) The relationship between the responsivity (R) and bias voltage of the ε-Ga_2_O_3_-based photodetectors under various light intensities. (**c**) The relationship between the detector responsivity (R) and external quantum efficiency (EQE) as a function of light intensity.

As shown in Figure 7a, the dark current is lower than 1 pA, and the photocurrent is higher than 1 nA after light is applied. The photo-to-dark current ratio (PDCR) is higher than three orders of magnitude under the 40 μW/cm^2^ irradiance of 254 nm UV light, and the photocurrent gradually increases with the light irradiance intensity. This indicates that ε-Ga_2_O_3_ has a strong detection ability in a wide range of light intensities. As the bias voltage increases, the PDCR rises and exceeds 10^6^, indicating an excellent solar-blind ultraviolet photodetection performance. The responsivity of an ideal photodetector is independent of the light irradiance, so the dependence of responsivity on voltage was calculated for different light intensities. As shown in Figure 7b, the responsivity–voltage curves obtained under various illumination intensities are almost overlapped. This demonstrates a stable responsivity of the ε-Ga_2_O_3_ photodetector across different light irradiance. The dependence of responsivity and external quantum efficiency (EQE) on optical power was further investigated under a 10 V bias Figure 7c. Both the responsivity and EQE decrease slightly with increasing power, indicating a mild influence of illumination intensity on the photoresponse.

## 4. Conclusions

Ultra-thin, pure-phase ε-Ga_2_O_3_ films with a preferred orientation were deposited in situ on sapphire substrates by thermal ALD at a relatively low temperature of 400 °C. Post-annealing studies show that the metastable ε-Ga_2_O_3_ phase could be stabilized upon post-annealing at temperatures up to 800 °C, and it transforms into the more stable β-Ga_2_O_3_ after annealing at 900 °C. The surface roughness of the ɛ-Ga_2_O_3_ does not change much after annealing, and the roughness slightly increases after the transformation into β-Ga_2_O_3_. The dark current of the solar-blind photodetector prepared based on ɛ-Ga_2_O_3_ is lower than 1 pA, and the PDCR is greater than 10^6^, which increases with the increase in light intensity. At the same time, the detector responsivity is almost independent of light intensity, which demonstrates a good day-blind detection performance and light intensity stability.

## Figures and Tables

**Table 1:** Summary and comparison of the β-Ga_2_O_3_ and ε-Ga_2_O_3_ thin films.

Properties	β-Ga_2_O_3_ Thin Film Growth	ε-Ga_2_O_3_ Thin Film Growth	Associated Challenges
Thermodynamics	Stable phase	Metastable phase	ε-phase is inherently prone to transform into β-phase
Growth Window	Broad	Extremely narrow	Requires highly precise parameter control
Temperature Control	Large-temperature growth window	Narrow-temperature growth window	Susceptible to phase transformation
Phase Purity	High-purity phase readily achievable	Highly prone to phase separation	Phase purity

## Data Availability

Data are contained within the article.
